# Titanium Dioxide Induces Apoptosis under UVA Irradiation via the Generation of Lysosomal Membrane Permeabilization-Dependent Reactive Oxygen Species in HaCat Cells

**DOI:** 10.3390/nano11081943

**Published:** 2021-07-28

**Authors:** In Young Kim, Tae Geol Lee, Vytas Reipa, Min Beom Heo

**Affiliations:** 1 Nano-Safety Team, Safety Measurement Institute, Korea Research Institute of Standards and Science (KRISS), Daejeon 34113, Korea; inyoungkim@kriss.re.kr (I.Y.K.); tglee@kriss.re.kr (T.G.L.); 2 Materials Measurement Laboratory, Biosystems and Biomaterials Division, National Institute of Standards and Technology, Gaithersburg, MD 20899, USA

**Keywords:** titanium dioxide nanoparticles (TiO_2_ NPs), ultraviolet A (UVA), phototoxicity, lysosomal membrane permeabilization (LMP), reactive oxygen species (ROS)

## Abstract

Titanium dioxide nanoparticles (TiO_2_ NPs) have wide commercial applications, owing to their small size; however, the biosafety of TiO_2_ NPs should be evaluated further. In this study, we aimed to investigate the cytotoxicity of TiO_2_ NPs in the presence and absence of ultraviolet A (UVA) irradiation in human keratinocyte HaCaT cells. TiO_2_ NPs did not significantly affect cell viability in the absence of UVA irradiation. Nonetheless, UVA-irradiated TiO_2_ NPs induced caspase-dependent apoptosis of HaCaT cells. Exposure of HaCaT cells to TiO_2_ NPs and UVA resulted in reactive oxygen species (ROS) generation and lysosomal membrane permeabilization (LMP); both effects were not observed in the absence of UVA irradiation. An analysis of the relationship between LMP and ROS, using CA-074 as a cathepsin inhibitor or NAC as an antioxidant, showed that LMP stimulates ROS generation under these conditions. These results imply that LMP-dependent oxidative stress plays a critical role in the UVA phototoxicity of TiO_2_ NPs in HaCaT cells.

## 1. Introduction

The potential toxicity of nanomaterials (NMs) to humans and the environment has been recognized since the dawn of nanotechnology [1]. This is because their physicochemical properties are highly dependent on small particle size, resulting in a high surface-to-volume ratio and enhanced transport across biological barriers [2]. Consequently, some materials, while benign in bulk form, become toxic when prepared in nano sizes [3].

Titanium dioxide nanoparticles (TiO_2_ NPs) are the highest volume-NM employed in water purification, paint, food additives, and personal care products, including sunscreens. There is a high probability that these nanoparticles (NPs) will come in contact with human skin and mucous membranes, subsequently entering the body [4]. Numerous studies have confirmed that the overall cytotoxicity of TiO_2_ NPs is rather low, compared with that of several widely produced NMs [5,6,7], and they are “generally regarded as safe (GRAS)” by regulatory agencies [8]. However, some countries lately have removed TiO_2_ from the GRAS designation owing to in vivo evidence of its potential toxicity [9]. In addition, in vitro studies with mammalian cells have shown that TiO_2_ NPs induce oxidative stress and apoptosis [10,11,12,13], suggesting that the safety profile of TiO_2_ NPs is still unclear.

A key determinant of NP toxicity is their ability to penetrate cellular membranes and their subcellular localization. NPs often accumulate in the lysosomes [14] via processes such as autophagy and endocytosis. Autophagy, a main catabolic pathway in mammalian cells, is involved in the elimination of foreign substances and cell degradation products by encapsulating them with a double membrane, thus forming the autophagosomes and subsequently fusing with lysosomes. Following their fusion, substances in autophagosomes are digested by lysosomal hydrolytic enzymes. Impairment of this process can lead to a variety of human diseases, and at the molecular level, it is linked to the damage of specific autophagy system components such as lysosomes. The uptake and accumulation of positively charged polystyrene nanoparticles have been reported to cause progressive lysosomal alterations, from early mild lysosomal membrane permeabilization (LMP), followed by lysosomal expansion and extensive LMP [13] after prolonged exposures. Substantial lysosomal damage leads to cathepsin release, thereby inducing cell apoptosis and necrosis [14,15,16]. Exposure to TiO_2_ NPs of various sizes for 24 h has been found to upregulate autophagic flux, lysosomal dysfunction, and membrane permeabilization, whereas a 72 h exposure abrogates autophagic flux [17,18].

Interest in TiO_2_ NPs is mostly owing to their photocatalytic activity, which is a major contributor to the toxicological effects of TiO_2_ NPs when they interact with biomolecules [19,20]. Phototoxicity is triggered by adverse chemical reactions supported by photocatalytically active materials under illumination. The principal cause of cell death mediated by photoactivated TiO_2_ NPs is the oxidative damage of proteins, nucleic acids, and lipids caused by excessive reactive oxygen species (ROS) generation when TiO_2_ NP absorb UV light and the excited charge carriers react with oxygen and/or water [21]. Therefore, the safety of TiO_2_ NPs is highly dependent on lighting conditions during their human and environmental exposure [22].

Due to the lack of clarity regarding the effects of the photocatalytic activity of TiO_2_ NPs on the human body, some regulators have restricted their use in cosmetic applications, including sunscreen [23]. Photocatalytic activity is a well-established physicochemical property of TiO_2_ NPs and is directly related to cellular toxicity under ultraviolet (UV) irradiation [24,25]. These properties also serve as a basis for the development of photodynamic therapies, as TiO_2_ NPs under UVA irradiation (310 nm < λ < 390 nm) induce cell death in colon carcinoma, melanoma, cervix adenocarcinoma, fibroblasts, and keratinocytes [26,27,28,29,30,31].

Considering the increasing interest in the nanomedical application of photoexcited TiO_2_ NPs and concerns about their safety in personal care products, the mechanistic aspects of cell impairment have lately become a focus of experimental studies [32,33]. Besides the production of ROS and ensuing oxidative stress as well-established underlying factors for the adverse biological reactions, other attributes such as NP subcellular localization may alter the phototoxic effects of TiO_2_ NPs. Photo-induced damage to the endoplasmic reticulum often leads to cell death by autophagy [34]. A significant increase in the number of autophagic vesicles, along with the degradation of the endoplasmic reticulum, but no inflammation, in TiO_2_-exposed human bronchial epithelial cells, has been reported by Yu et al. [35]. Moreover, TiO_2_ NP-generated ROS can also promote the uncoupling of the mitochondrial respiratory chains, resulting in excess intracellular ROS production from the superoxide anion [36]. Other studies have shown that UV irradiation aggravates LMP, which leads to the intrinsic apoptosis of melanocytes [37,38] and cell necroptosis by hindering autophagosome–lysosome fusion [39]. These findings suggest that the role of photoexcitation in autophagy and lysosomal damage is not well established [40].

In this study, we comprehensively investigated the biological mechanism of phototoxicity by TiO_2_ in HaCaT cells and found evidence of the lysosomal membrane damage that facilitates intracellular ROS generation. We focused on the combined effects of TiO_2_ NP uptake and resulting UVA sensitization on lysosomal membrane integrity. We found evidence that major lysosomal destabilization occurs only when accumulated TiO_2_ NPs are photoexcited by UVA. Consequently, the loss of lysosomal membrane integrity is essential for TiO_2_ NPs and UVA-induced ROS surge. Moreover, our data strongly suggest that caspase-dependent apoptosis is a key mechanism of HaCaT cell death induced by their exposure to internalized and photoexcited TiO_2_ NPs.

## 2. Materials and Methods

### 2.1. Chemicals

z-VAD-fmk was purchased from R&D system (Minneapolis, MN, USA). 5-(and-6)-chloromethyl-2′,7′-dichlorodihydrofluorescein diacetate, acetyl ester (CM-H_2_DCF-DA) was purchased from Thermo Fisher Scientific (Waltham, MA, USA). Chlorpromazine hydrochloride, propidium iodide, acridine orange (AO), hydrogen peroxide (H_2_O_2_), N-acetyl-L-cysteine (NAC), and (L-3-trans-(propylcarbamyl)oxirane-2-carbonyl)-L-isoleucyl-L-proline (CA-074) were purchased from Sigma-Aldrich (St. Louis, MO, USA).

### 2.2. Cell Culture

Human keratinocyte HaCaT cells (Cell Line Services, Eppelheim, Germany) were maintained in Dulbecco’s modified Eagle medium containing 10% fetal bovine serum and 1% penicillin–streptomycin (all obtained from GIBCO-BRL, Grand Island, NY, USA). The cells were incubated under 5% CO_2_ at 37 °C.

### 2.3. Preparation and Characterization of TiO_2_ NP Suspension

TiO_2_ NPs (AEROXIDE^®^ TiO_2_ P25) were obtained from Evonik Industries AG (Essen, Germany). They were dispersed in a complete medium according to the National Institute of Standards and Technology (USA) special publication 1200–4 [41]. Particle size distribution of TiO_2_ NPs was determined by dynamic light scattering (DLS; ZEN5600, Malvern Panalytical, Worcestershire, England). Zeta potential was measured using the same equipment mentioned above.

### 2.4. Treatment with TiO_2_ NPs and UVA

**Figure 1:**
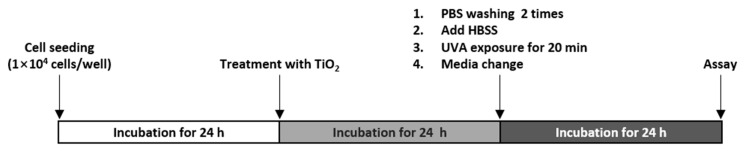
Outline of the experimental procedure.

A graphic summary of the experimental procedure is shown in Figure 1. HaCaT cells were plated in two 96-well plates and then incubated for 24 h. On a subsequent day, the surface-adhered cells were exposed to 25–200 μg/mL TiO_2_ NPs for 24 h. The treated cells were washed twice with warm phosphate-buffered saline (PBS) (Sigma-Aldrich, St. Louis, MO, USA) to separate extracellular TiO_2_ NPs. While one plate was directly irradiated with UVA for 20 min (light dose P = 5 J/cm^2^, exposure group), the other plate was protected from UVA exposure by covering with foil (dark group). Following post-irradiation stabilization for 20 min, HBSS was replaced with a complete medium, and the cells were incubated for 24 h.

### 2.5. Cell Viability Assay

The cells were seeded in 96-well plates and treated as described above. The CellTiter-Glo Luminescent Cell Viability kit (Promega, Madison, WI, USA) was employed to assess cell viability, according to the manufacturer’s instructions. The IC_50_, the concentration at which cell growth is inhibited by 50%, compared with the untreated control, was estimated using GraphPad Prism software 7.0 (GraphPad Software Inc., San Diego, CA, USA). Since treatment with 200 μg/mL TiO_2_ in the dark did not cause more than 50% viability decrease in HaCaT cells, the IC_50_ was estimated from the available viability data using GraphPad Prism software 7.0.

### 2.6. Measurement of Lactate Dehydrogenase Release

A lactate dehydrogenase (LDH) detection kit (CytoTox96^®^ Non-Radioactive Cytotoxicity Assay, Promega) was used to evaluate the integrity of the cell membrane. The release of LDH into the culture medium indicates irreversible cell death due to cell membrane damage [42,43]. After treating the cells as mentioned above, 50 µL of the supernatant was collected and tested according to the manufacturer’s instructions. LDH leakage was calculated using the following equation. LDH_min_ is the LDH leakage value of untreated cells. LDH_max_ is the LDH leakage value of cells treated with 2% Triton-X100 (positive control) for 10 min.

(1) $$\mathrm{LDH}\;\mathrm{leakage}\;(\%\;\mathrm{of}\;\max\;\mathrm{LDH}\;\mathrm{release})=\frac{\left(\mathrm{LDHexp}-\mathrm{LDHmin}\right)}{\left(\mathrm{LDHmax}-\mathrm{LDHmin}\right)}\;\times 100$$

### 2.7. Cell Cycle Analysis

DNA fragmentation is a hallmark of apoptosis [44], and therefore, apoptotic cells eventually develop a deficit in DNA content. In DNA content histograms, apoptotic cells often form characteristic “sub-G1” or “hypodiploid” peaks [45,46,47]. HaCaT cells were seeded at a density of 2 × 10^5^ cells/well in 6-well plates and incubated for 24 h. After treatment, HaCaT cells were harvested and fixed in 70% (*v*/*v*) ethanol. To evaluate the DNA content, cells were treated with RNase and stained with propidium iodide. The cells were analyzed using a FACSVerse flow cytometer (BD Biosciences, San Jose, CA, USA). The data were analyzed using FlowJo (version X, BD Biosciences).

### 2.8. Apoptosis Determination

Cells were seeded at a density of 2 × 10^5^ cells/well in 6-well plates and treated with TiO_2_ and UVA. The cells were incubated with 5 μL of Annexin V and 5 μL of propidium iodide (PI) for 15 min at room temperature in the dark, according to the manufacturer’s instructions (BD), and then subjected to flow cytometry to measure the apoptosis rate (%).

### 2.9. Cell Lysates and Western Blotting

HaCaT cells were seeded in 60 mm dishes and treated as mentioned above. The treated cells were scraped and harvested using PBS. After discarding the supernatant, the cell pellets were suspended in 2× Laemmli sample buffer with 5% β-mercaptoethanol (Bio-Rad, Hercules, CA, USA), and boiled for 7 min. The lysates were separated by 4–15% SDS–PAGE and transferred onto an Immobilon membrane (Millipore, Burlington, MA, USA). In this study, the following primary antibodies were used: PARP1 (1:1000, ab32071, Abcam, Cambridge, UK) and β-actin (1:5000, #3700, Cell Signaling Technology, Beverly, MA, USA). The secondary antibodies used were horseradish peroxidase (HRP)-conjugated goat anti-rabbit immunoglobulin G (IgG) and goat anti-mouse IgG (Invitrogen, Waltham, MA, USA). Bands were developed using the WesternBright ECL HRP substrate (Advansta, CA, USA). Quantification of the immunoblots was performed using Image J (NIH, MD, USA).

### 2.10. Transmission Electron Microscopy

The cells were prefixed in Karnovsky’s solution (1% paraformaldehyde, 2% glutaraldehyde, 2 mM calcium chloride, and 0.1 M cacodylate buffer, pH 7.4) for 2 h and washed with cacodylate buffer. Postfixing was carried out in 1% osmium tetroxide and 1.5% potassium ferrocyanide for 1 h. After dehydration with 50–100% alcohol, the cells were embedded in Poly/Bed 812 resin (Pelco, Redding, CA, USA), polymerized, and observed under an electron microscope (EM 902A; Carl Zeiss, Oberkochen, Germany).

### 2.11. Lysosomal Integrity Assay

As AO is a lysosomotropic dye that exhibits a red fluorescence under acidic conditions and green fluorescence in the non-acidic environment, therefore lysosomal acidification can be observed using the green/red fluorescence ratio in live cells [48]. We adopted the method described by Sun and Gan [49]. For fluorescence microscopy, HaCaT cells were seeded in 12-well plates and treated as mentioned above. After washing with PBS, the cells were stained with 5 μg/mL AO in DMEM for 15 min in the dark at 37 °C. Next, cells were washed with PBS again and further visualized using a fluorescence microscope (Leica Microsystems, Wetzlar, Germany). To quantitate fluorescence using a microplate reader, the cells were seeded in 96-well plates and allowed to attach to the plate for 24 h. The cells were stained with 5 μg/mL AO for 15 min. After washing with PBS, the cells were subsequently treated with TiO_2_ NPs and subjected to UVA irradiation. The treated cells were incubated for 8 h and then washed with HBSS. Fluorescence was measured at an excitation wavelength of 485 nm and two emission wavelengths of 530 (green AO) and 620 nm (red AO). Normal lysosomal integrity = total red fluorescence intensity of normal lysosomes / total green fluorescence intensity of normal lysosomes. Lysosomal integrity = total red fluorescence intensity / (total green fluorescence intensity × normal lysosomal integrity).

### 2.12. Measurement of ROS

When CM-H_2_DCF-DA is oxidized by ROS such as H_2_O_2_ and free radicals, it can be detected by monitoring the increase in fluorescence [50]. The TiO_2_ treatment procedure is described above. The treated cells were loaded with 10 µM CM-H_2_DCF-DA for 30 min in the dark at 37 °C and washed with HBSS. Fluorescence of the cells was measured using a microplate reader (λ_ex_ = 495 nm/λ_em_ = 525 nm) and also observed by fluorescence microscopy.

### 2.13. Inhibitor Study

To investigate the relationship between LMP, ROS generation, and cell death, we used CA-074, a cathepsin B inhibitor, and NAC, a well-known antioxidant. When the medium was replaced after TiO_2_ NP treatment and UVA irradiation, each inhibitor was added to the media prior to subsequent cell incubation.

### 2.14. Statistical Analysis

All data are presented as mean ± standard error of the mean (SEM) or one standard deviation (SD) of at least three separate experiments. GraphPad Prism 7.0 software was used to perform statistical analyses. The normality of data was assessed using Kolmogorov–Smirnov test, and equal variance was assessed using Bartlett’s test. For normally distributed data, statistical differences were determined using the analysis of variance, followed by Bonferroni’s multiple comparison test. If the data were not normally distributed, Kruskal–Wallis test was performed followed by Dunn’s test.

## 3. Results

### 3.1. Characterization of the TiO_2_ NP Suspension Employed for Cellular Exposure

**Figure 2:**
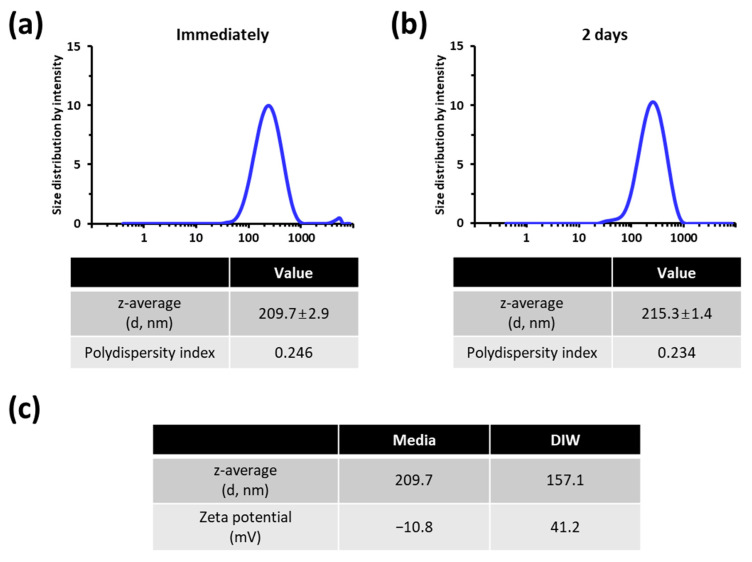
Characterization of TiO_2_ NP dispersion in the cell growth media. TiO_2_ NPs were dispersed at 1 mg/mL in DMEM. Size was measured by DLS immediately (**a**) and 2 days (**b**) after dispersion. (**c**) comparison TiO_2_ NPs dispersions in DIW and media. Size and zeta potential were measured immediately following the dispersion procedure.

DLS was performed to determine the particle size distribution of the TiO_2_ NP suspension (Figure 2). After dispersion in media, the z-average size of TiO_2_ NPs was 209.7 ± 2.9 nm (diameter, intensity weighted) with a polydispersity index of 0.246 (Figure 2a). In addition, the stability of the TiO_2_ NP suspension during the test duration was tested. The particle size distribution profile was almost constant for 2 days (Figure 2b), indicating that the stability of the TiO_2_ NP suspension was acceptable.

Since NP size distribution can undergo significant changes when transferred to environments used for biological studies [51], we characterized the dispersions of TiO_2_ NPs in deionized water (DIW) and media by measuring size distributions and zeta potential values (Figure 2c). As expected, the z-average size of TiO_2_ NPs was larger in the media (209.7 nm) than in DIW (157.1 nm) (Figure 2c). The zeta potential was 41.2 mV in DIW and −10.8 mV in media (Figure 2c). These results suggest that the hydrodynamic size and zeta potential TiO_2_ NPs both are affected due to the interaction with media components.

### 3.2. UVA Irradiation Induces Toxicity in HaCaT Cells Treated with TiO_2_ NPs

**Figure 3:**
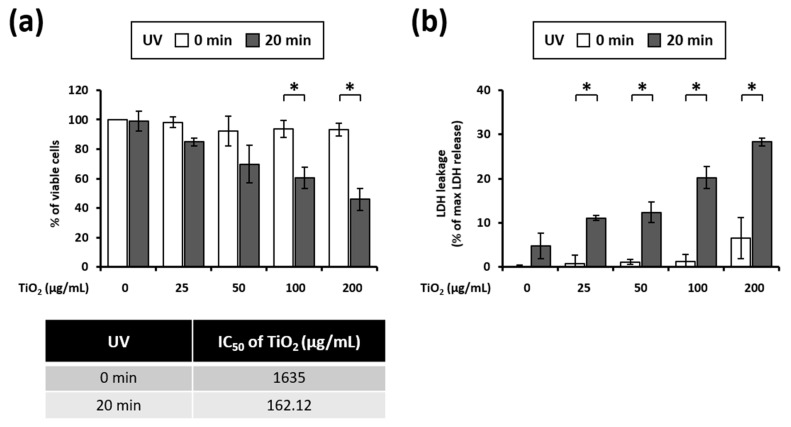
Effects of UVA exposure on HaCaT cells treated with TiO_2_ NPs: (**a**) cellular viability was evaluated using the CellTiter-Glo^®^ Luminescent cell viability assay; (**b**) lactate dehydrogenase (LDH) released by the cells was detected. Data are presented as mean ± SEM (*n* = 3). * *p* < 0.05, compared with dark conditions.

The 3T3 cell line is the most used cell line to study the effect of phototoxicity. These cells have several advantages, including availability, easy and inexpensive cultivation, as well as reproducibility. Recently, Svobodova et al. [52] reported that human keratinocyte HaCaT cells are also appropriate for in vitro phototoxicity testing. We have investigated how the viability of HaCaT cells is affected by a combined action of TiO_2_ NPs exposure and UVA irradiation. Cell viability measurement demonstrated that HaCaT cells were largely unaffected to both TiO_2_ exposure (up to 200 µg/mL) and UVA irradiation (up to 5 J/cm^2^) treatment if applied separately (Figure 3a). However, the combination of TiO_2_ NPs and UVA irradiation markedly reduced the viability of HaCaT cells (Figure 3a). To quantify TiO_2_ NP phototoxicity, we estimated the NP concentration at which 50% of cell growth (IC_50_ value) is inhibited with and without UVA irradiation. The IC_50_ of the samples was reduced 10-fold with UVA irradiation, compared with that of nonirradiated samples (Figure 3a). To confirm cellular damage, we have performed the LDH assay. TiO_2_ NPs alone at lower concentrations (up to 100 µg/mL) did not induce a noticeable LDH release. However, the LDH activity was upregulated in cells that were subjected to the combined treatment with UVA compared to those treated with TiO_2_ NPs alone (Figure 3b). Taken together, these results clearly show that a combination of UVA irradiation and TiO_2_ NP exposure significantly enhances the cytotoxicity.

### 3.3. UVA Irradiation Facilitates the Apoptosis of HaCaT Cells Treated with TiO_2_ NPs

**Figure 4:**
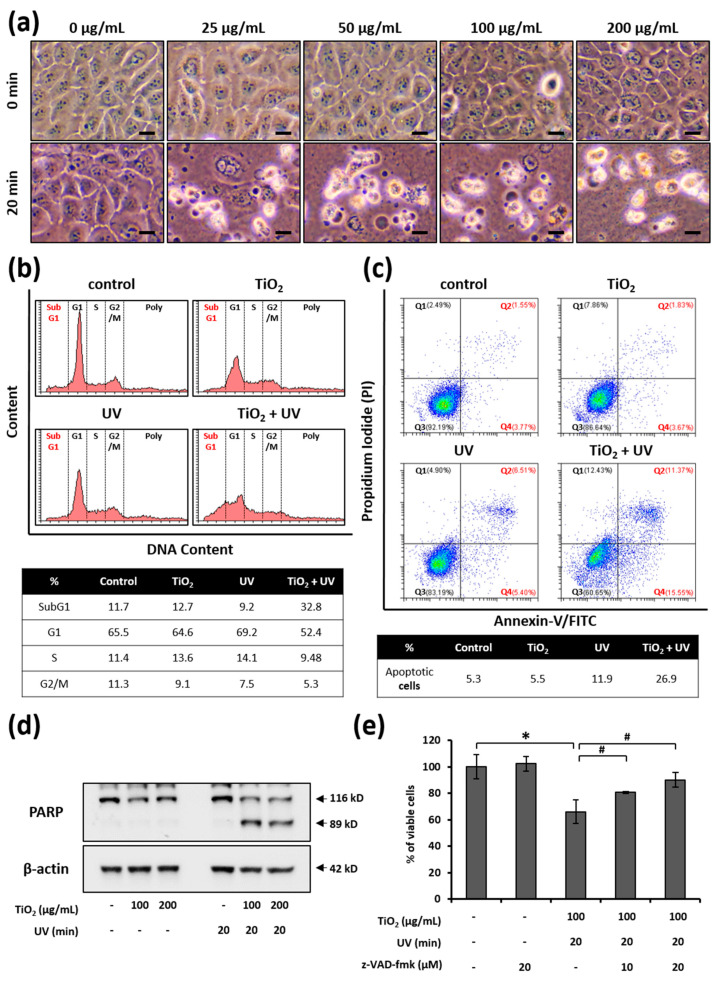
Apoptosis induction by the combination of TiO_2_ NPs and UVA: (**a**) the treated cells were observed by bright-field microscopy. Scale bars correspond to 20 µm; (**b**) HaCaT cells were treated with a combination of TiO_2_ NPs and UVA. The DNA content was measured by flow cytometry after PI staining; (**c**) the treated cells were harvested and apoptosis was analyzed using the Annexin V-FITC Apoptosis Detection Kit by flow cytometry. The cells were classified as healthy cells (Q3; Annexin V−, PI−), early apoptotic cells (Q4; Annexin V+, PI−), late apoptotic cells (Q2; Annexin V+, PI+), and damaged cells (Q1; Annexin V−, PI+); (**d**) lysates were extracted from cells treated with TiO_2_ NPs and/or UVA. Western blotting of PARP was performed. β-Actin was used as the loading control; (**e**) the cells were treated with TiO_2_ NPs for 24 h and subsequently irradiated with UVA for 20 min. HBSS was replaced with the complete medium containing z-VAD-fmk, and the cells were incubated for 24 h. Cellular viability was determined using the CellTiter-Glo^®^ Luminescent cell viability assay. Data are presented as mean ± SEM (*n* = 3). * *p* < 0.05, compared with the control; # *p* < 0.05, compared with the combination of TiO_2_ NPs and UVA.

Next, we examined the morphological changes in cells that occurred during the combined TiO_2_ NP and UVA treatment. The cells treated with TiO_2_ NPs or UVA irradiation alone remained attached to the substrate and largely retained their shape. In contrast, cells treated with both TiO_2_ NPs and UVA irradiation detached from the plate surface and became smaller and spherical (Figure 4a). Given that cell shrinkage, or cell volume loss, is often an indication of apoptosis [41], we verified whether the cell death induced by the combined treatment with TiO_2_ NPs and UVA was apoptotic in nature. We examined the changes in DNA content following treatment with TiO_2_ NPs and UVA irradiation (Figure 4b). Only 12.7% of HaCaT cells treated with TiO_2_ NPs alone for 24 h were in the sub-G1 phase, and there was no dramatic change in the DNA content, compared to the control group. However, treatment with both TiO_2_ NPs and UVA increased the cell population in sub-G1 to 32.8%. Simultaneously, G1, S, and G2/M populations were also smaller. Next, we detected the population of apoptotic cells using Annexin-V binding and PI staining. While the population of apoptotic cells (Q2 + Q4) did not change under treatment with TiO_2_ alone compared to the control group, it increased 6.6% after UVA irradiation alone. In addition, the combination treatment with TiO_2_ NPs and UVA sharply increased the proportion of apoptotic cells to 26.9%. These results provide further evidence that a combination of TiO_2_ and UVA leads to HaCaT cell death via the apoptotic pathway.

Since caspases are known as executioners in apoptosis [53], we also examined whether the TiO_2_ NP and UVA-induced cell death was mediated by caspase activation. We found no evidence that poly (ADP-ribose) polymerase (PARP), a caspase substrate, was processed following the separate treatments of HaCaT cells with TiO_2_ NPs or UVA (Figure 4c). However, when both TiO_2_ NPs and UVA were applied, PARP was progressively processed (Figure 4c). To ascertain whether caspases are important in apoptosis induced by the combination of TiO_2_ NPs and UVA, we investigated the effect of the pan-caspase inhibitor z-VAD-fmk on cell viability. The treatment of HaCaT cells with z-VAD-fmk blocked co-treatment-induced cell death in a dose-dependent manner (Figure 4d). Taken together, these results confirm that TiO_2_ NPs under UVA irradiation induces the caspase-dependent apoptosis in HaCaT cells.

### 3.4. TiO_2_ NPs Accumulate in Lysosomes

**Figure 5:**
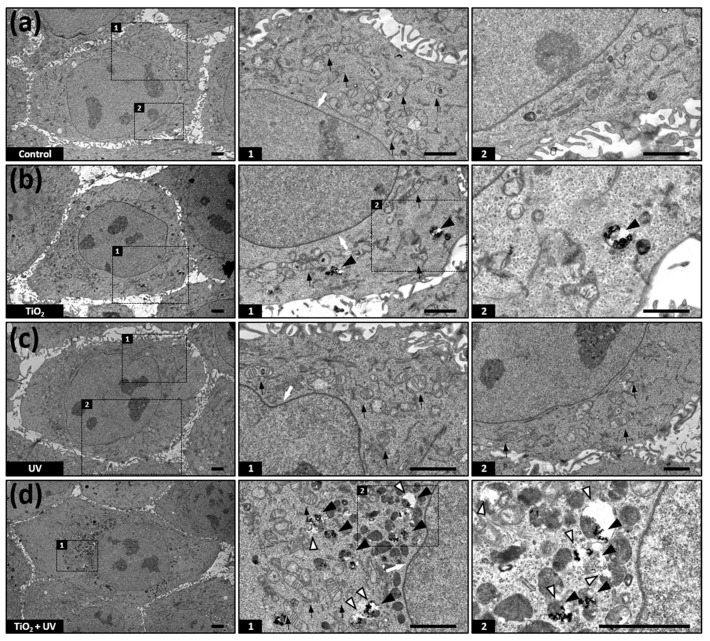
Transmission electron microscopy of HaCaT cells treated with TiO_2_ NPs and UVA: (**a**) untreated cells; (**b**) cells treated with TiO_2_ NPs alone; (**c**) cells irradiated with UVA for 20 min; (**d**) cells cotreated with TiO_2_ NPs and UVA. Scale bars correspond to 2 µm. Black arrowhead, phagosome-like structures; black arrow, mitochondria; white arrow, nuclei; white arrowhead, ruptured lysosome.

To observe the cellular uptake of TiO_2_ NPs and structural changes in the cellular organelles in TiO_2_ NP- and UVA-treated cells, we have employed electron microscopy (Figure 5). At first we have confirmed the cellular uptake of TiO_2_ NPs. TiO_2_ NPs were not detected in untreated or only UVA-irradiated cells (Figure 5a,c). After treatment with TiO_2_ NPs alone and in combination with UVA, TiO_2_ NPs aggregates were observed in phagosome-like structures (black arrowhead) but not in the nuclei (white arrow) or mitochondria (black arrow) (Figure 5b,d). Next, we observed alterations in the cellular organelle structures. The organelles did not substantially change except when treated with both TiO_2_ NPs and UVA (Figure 5a,c). Remarkably, the mitochondrial structure remained almost unaltered in all of the imaged samples (Figure 5, black arrow). Moreover, we found that the combination of TiO_2_ NPs and UVA increased the number of phagosomal and lysosomal structures containing TiO_2_ NPs (Figure 5d, black arrowhead). These membranes were noticeably ruptured (Figure 5d, white arrowhead).

### 3.5. Combination of TiO_2_ NPs and UVA Induces LMP

**Figure 6:**
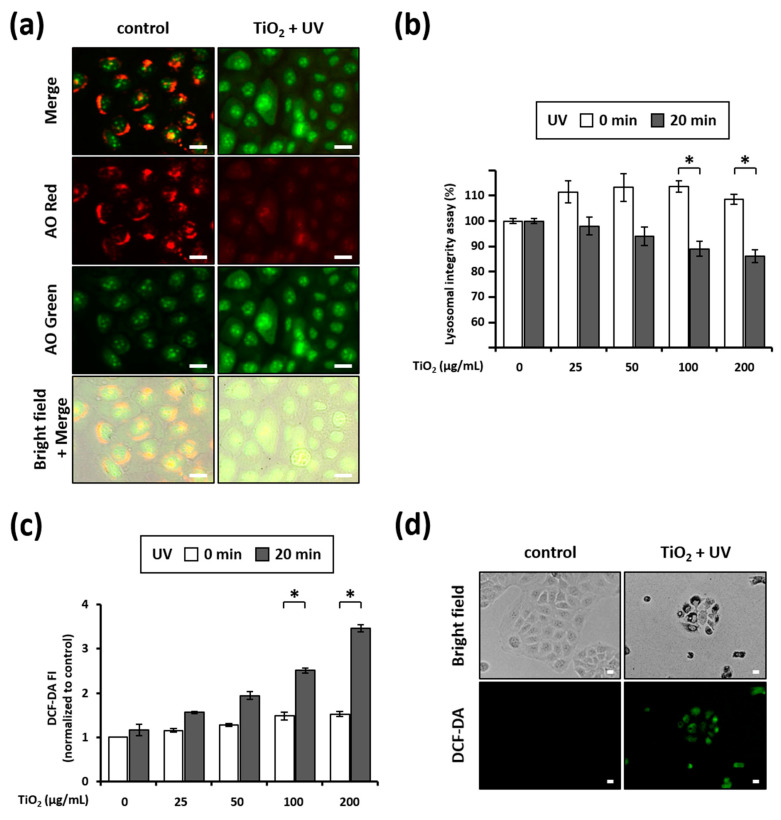
LMP induction and ROS generation by the combination of TiO_2_ NPs and UVA: (**a**) the treated cells were stained with AO and then observed by fluorescence microscopy. Scale bars correspond to 10 µm; (**b**) after AO staining, the cells were treated with TiO_2_ NPs and UVA for 8 h. The cells were subjected to microplate reading. Data are presented as mean ± SD (*n* = 8). * *p* < 0.05, compared with dark conditions; (**c**,**d**) the treated cells were stained with CM-H_2_DCF-DA. The cells were subjected to microplate reading: (**c**) data are presented as mean ± SD (*n* = 6). * *p* < 0.05, compared with dark conditions; (**d**) cells were observed by fluorescence microscopy. Scale bars correspond to 20 µm.

As our results suggested that lysosomal membranes were altered by the combination of TiO_2_ NPs and UVA (e.g., ruptured membranes in Figure 5), we tested the lysosomal integrity of HaCaT cells by staining them with AO. While red fluorescence was strong, green fluorescence was rather weak in the untreated cells. However, the cells treated with TiO_2_ NPs and UVA mostly showed diminished red fluorescence and increased green fluorescence (Figure 6a), indicating that the lysosomes were severely damaged. Next, we quantified the lysosomal integrity using a microplate reader. The integrity of the lysosomes in cells treated with the combination of TiO_2_ NPs and UVA decreased to 89.04 ± 3.98% and 86.02 ± 4.01% following the incubation with 100 and 200 µg/mL TiO_2_ NPs, respectively, relative to lysosomes in untreated cells (100%) (Figure 6b). These results suggest that lysosomal membranes are damaged during HaCaT cell death triggered by the combined treatment with TiO_2_ NPs and UVA.

### 3.6. Combination of TiO_2_ NPs and UVA Induces ROS Generation

The oxidative damage of cellular molecules caused by excessive ROS production is known as a major factor driving the phototoxicity of TiO_2_ NPs [26,27,28,29,30]. In this study, we assayed intracellular ROS production using CM-H_2_DCF-DA dye. Independent treatment with TiO_2_-NPs and UVA generated rather low levels of intracellular ROS in a concentration-dependent manner (Figure 6c), whereas the combination of TiO_2_ NPs and UVA significantly increased intracellular ROS. This was particularly noticeable in the cells treated with 200 µg/mL TiO_2_ NPs, wherein, the intracellular ROS level increased by approximately 2.3 fold following treatment with UVA irradiation, compared with level in the non-irradiated sample (Figure 6c). Again, fluorescence microscopy images confirmed that TiO_2_ NPs and UVA noticeably boosted the ROS levels when combined (Figure 6d). These results confirm that intracellular ROS production accompanies HaCaT cell death caused by the combination of TiO_2_ NPs and UVA.

### 3.7. Relationship among LMP, ROS Generation, and Cell Death in HaCaT Cells Treated with TiO_2_ NPs and UVA

**Figure 7:**
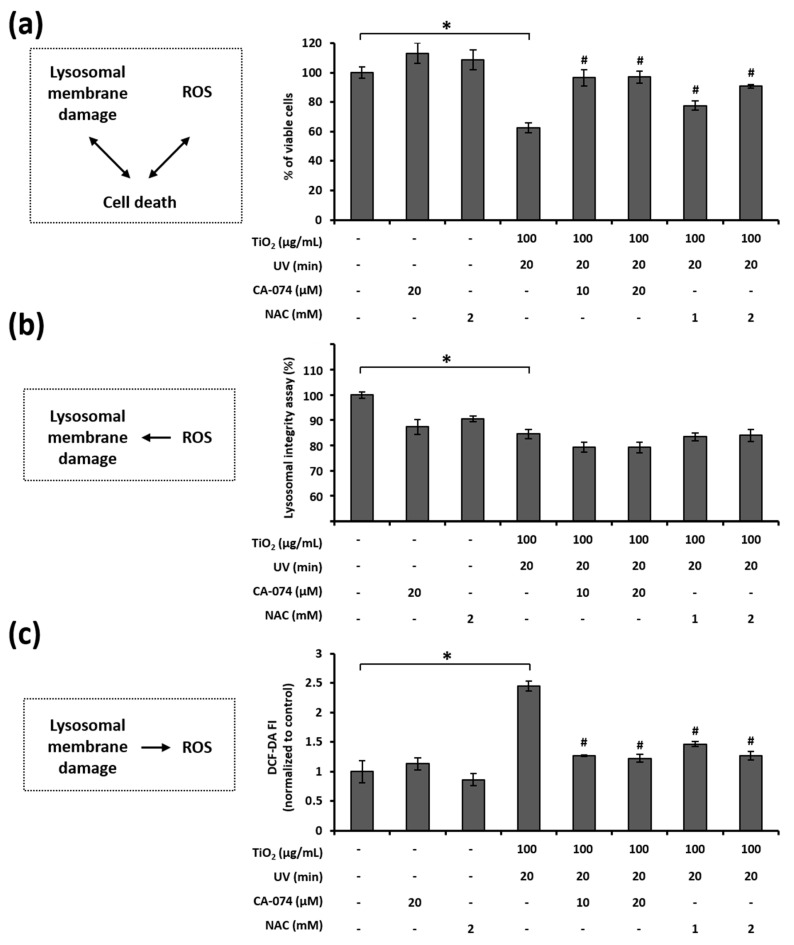
Interaction among cell death, LMP, and ROS in HaCaT cells treated with TiO_2_ NPs and UVA: (**a**) the cells were treated with TiO_2_ NPs for 24 h and subsequently irradiated with UVA for 20 min. HBSS was replaced with the complete medium containing CA-074 or NAC, and the cells were incubated for 24 h. Cellular viability was evaluated using the CellTiter-Glo^®^ Luminescent cell viability assay; (**b**) after AO staining, the cells were treated with TiO_2_ NPs, UVA, and inhibitors for 8 h. The cells were then subjected to microplate reading; (**c**) the treated cells were stained with CM-H_2_DCF-DA. The cells were subjected to microplate reading. Data are presented as mean ± SD (*n* = 8). * *p* < 0.05, compared with untreated cells; # *p* < 0.05, compared with the combination of TiO_2_ NPs and UVA.

We examined the link between lysosomal membrane damage, ROS generation, and cell death induced by the combination of TiO_2_ NPs and UVA. First, we assessed the role of the lysosomal membrane damage and ROS generation in cell death by means of CA-074, a cathepsin B inhibitor, and NAC, a well-known antioxidant. The treatment of HaCaT cells with CA-074 or NAC effectively inhibited the cell death, as caused by the combined treatment with TiO_2_ NPs and UVA (Figure 7a). This result implies that both lysosomal membrane damage and ROS generation are critical contributors to cell death under these conditions.

Next, we investigated the relationship between lysosomal membrane damage and ROS generation. ROS are known to induce LMP, especially when highly reactive hydroxyl radicals are produced in the lysosomes [54,55,56]. Therefore, we wanted to determine whether the loss of lysosomal membrane integrity occurs when ROS are produced in response to the combined TiO_2_ NPs and UVA treatment. Unexpectedly, neither CA-074 nor NAC were effective in preserving the lysosomal membrane integrity in cells treated with TiO_2_ NPs and UVA (Figure 7b). This result indicates that ROS increase was not the primary reason for the decline in lysosomal membrane integrity. Furthermore, as LMP may increase the generation of intracellular ROS via the release of free iron into the cytosol [57,58,59], we evaluated whether ROS can be generated due to LMP. Notably, both CA-074 and NAC attenuated ROS generation in response to the combined treatment with TiO_2_ NPs and UVA (Figure 7c). This result suggests that the loss of lysosomal membrane integrity is vital for TiO_2_ NP and UVA-induced ROS generation. Taken together, these results show that the combination of TiO_2_ NPs and UVA induces cell death via LMP-dependent ROS generation in HaCaT cells.

## 4. Discussion

TiO_2_ NPs are among the most widely used nanomaterials in paints, plastics, cosmetics, and personal care products; they are also used as food additives and drug delivery agents [60,61]. They are widely used owing to their biocompatibility, high chemical stability, photocatalytic properties, and pronounced sensitivity to heat and magnetism [62,63,64]. The TiO_2_ NPs are being explored as potential candidates for degrading organic pollutants [65] and inactivating microorganisms owing to their photocatalytic activity [60,66]. However, these photocatalytic properties were reported to trigger oxidative damage, cellular structure destruction, key protein inactivation, and DNA damage, leading to cell apoptosis or necrosis [67,68,69,70]. To support their safe and efficient use, it is essential to identify mechanisms underlying the photocatalytic activity and phototoxicity of TiO_2_ NPs. In this study, we evaluated the phototoxicity of TiO_2_ NPs on human skin keratinocytes subjected to UVA irradiation and investigated the mechanistic aspects of the resulting cell death.

As shown in Figure 3, TiO_2_ NPs exerted UVA-induced and dose-dependent toxicity in HaCaT cells. As cell viability decreases after TiO_2_ NP and UVA treatment, we explored the mode of death of these cells. The photocatalytic activity of TiO_2_ NPs can induce apoptosis and necrosis through various mechanisms [67,68,70,71,72]. Apoptosis is a form of programmed cell death characterized by caspase activation, cell contraction, apoptotic body formation, exposure outside the cell membrane, chromatin condensation, and DNA fragmentation [73,74,75]. Our data show that the DNA sub-G1 population increases following combined treatment with TiO_2_ NPs and UVA (Figure 4b). In addition, PARP, a substrate of caspase-3, is cleaved and z-VAD-fmk, a pan-caspase inhibitor, increases the viability in HaCaT cells co-treated with TiO_2_ NPs and UVA (Figure 4c,d). These results suggest that the TiO_2_ NPs and UVA-induced cell death is caused by the caspase-dependent apoptosis.

We detected TiO_2_ NPs inside HaCaT cells using transmission electron microscopy; this confirmed that the phototoxicity observed in this study is induced by the intracellular accumulation of TiO_2_ NPs. Consistent with the results of Tucci et al. [76], TiO_2_ NPs accumulated in the phagosomal and lysosomal structures of the cells but not in the nuclei or mitochondria (Figure 5b,d). Remarkably, we observed lysosomal membrane rupture in HaCaT cells treated with TiO_2_ NPs and UVA (Figure 5d). The maintenance of lysosomal membrane integrity is vital for cell viability because ruptured lysosomal membranes induce the leakage of various digestive enzymes into the cytosol, resulting in membrane trafficking defects, abnormal energy metabolism, and cell death [77]. Recently, lysosomal damage has been suggested as a key mechanistic element of nanoparticle toxicity [78,79,80] because most endocytosed nanoparticles were found to accumulate in lysosomal compartments. In our study, we evaluated the lysosomal membrane integrity by AO staining and determined that combined treatment with TiO_2_ NPs and UVA damaged the lysosomal membranes in HaCaT cells (Figure 6a,b). In addition, CA-074, a cathepsin B inhibitor that protects against lysosomal rupture [81], prevented cell death induced by the combination of TiO_2_ NPs and UVA (Figure 7a). These results demonstrate that LMP is imperative for TiO_2_ NP and UVA-induced cell death.

As ROS-mediated LMP is the prevailing mechanism [82], we examined the link between LMP and ROS generation when HaCaT cells are exposed to TiO_2_ NPs and UVA. First, we found that TiO_2_ NPs subjected to UVA irradiation upregulated ROS generation in HaCaT cells (Figure 6c,d). In addition, the inhibition of ROS increased cell viability (Figure 7a), confirming the critical regulatory role of ROS in the progression of HaCaT cell apoptosis. Next, we tested lysosomal membrane integrity and ROS production in cells treated with the cathepsin B inhibitor CA-074 and antioxidant NAC. Interestingly, while CA-074 prevented ROS generation, NAC did not prevent the loss of lysosomal membrane integrity (Figure 7b,c). Although several factors and chemicals have been reported to induce LMP and the associated cell death [83,84,85], it is not clear whether lysosomal leakage leads to oxidative stress during apoptotic cell death driven by the lysosomal pathway. Our study, for the first time, proves that lysosomal destabilization by TiO_2_ NPs and UVA induces cellular ROS generation and oxidative stress.

Recent reports showed that besides TiO_2_ NPs other NPs, including ZnO, Au, and Ag NPs, exhibit photocatalytic activity [86]. In particular, the phototoxicity of ZnO NPs is studied because they are often included in paints, cosmetics, and medical materials, along with TiO_2_ NPs. Wang et al. [87] have reported that the phototoxicity of ZnO NPs under UVA and visible light irradiation is related to oxidative DNA damage. As ZnO NPs and TiO_2_ have comparable bandgap energy (3.2 eV) and photocatalytic activity [88], they can be expected to cause a similar cellular toxicity. However, the important difference between these two materials is their chemical stability, as ZnO slowly dissolves under UV illumination, releasing metal ions, while TiO_2_ is chemically stable.

## 5. Conclusions

Previous studies have shown that ROS generation is the primary cause of the phototoxicity in cells treated with TiO_2_ under UVA irradiation. In this study, we examined the mechanism underlying the phototoxicity induced by TiO_2_ in HaCaT cells. We present evidence that TiO_2_ induces lysosomal membrane damage and subsequently stimulates ROS production. We focused on the effects of TiO_2_ NP uptake and UVA sensitization on lysosomal membrane integrity in HaCaT cells. We found that major lysosomal destabilization occurs when accumulated TiO_2_ NPs are photoexcited by UVA. Consequently, the loss of lysosomal membrane integrity is essential for TiO_2_ NPs and UVA-induced ROS surge. Taken together, our findings strongly suggest that caspase-dependent apoptosis is a key mechanism underlying HaCaT cell death, induced by their exposure to photoexcited TiO_2_ NPs.

Certain instruments and materials are identified in this paper to adequately specify experimental details. In no case does it imply endorsement by NIST or that it is necessarily the best product for the experimental procedure.

## Data Availability

All data are contained within the article.
